# Development, content validity and application feasibility of the data collection tool for forensic section in Iranian Integrated Road Traffic Injury Registry (IRTIR)

**Published:** 2019-08

**Authors:** Bahram Samadirad, Abdolrazagh Barzegar, Fereidun Ashrafian Bonab, Homayun Sadedeghi- Bazargani, Artin Kamali, Ommolbanin Abbasnezhad

**Affiliations:** ^*a*^Legal Medicine Research Center, Legal Medicine Organization, Tehran, Iran.; ^*b*^East Azarbaijan Legal Medicine General Office, Tabriz, Iran.; ^*c*^Road Traffic Injury Research Center, Tabriz University of Medical Science, Tabriz, Iran.

## Abstract

**Background:**

The present study was conducted as part of the national project on developing Integrated Road Traffic Registry (IRTIR). The aim of this study was to develop and assess application feasibility and content validity of data collection tool for forensic section in IRTIR.

**Methods:**

Data collection tools were developed by information retrieved from literature review as well as the current tool used for traffic fatalities in Forensic Medicine Organization (FMO) and discussion in expert panel. For assessment of content validity, 15 experts in various fields of expertise such as forensic medicine, emergency medicine and neurosurgery, expressed their ideas and gave comments along with scoring the items on relevance, clarity, necessity and feasibility. The Content validity was calculated by the content validity index (CVI) and modified KAPPA. In order to evaluate the application feasibility of the tool, the information for ten traffic fatal cases were collected by forensic medicine specialists using the developed tool.

**Results:**

The content validity of the data collection tool was confirmed after two rounds of assessment and improvement. It also was found to be feasible for use in forensic investigations. According to the CVI and modified kappa value above 0.87 for all items. The final tool included in various areas of etiologic and prevention-related information.

**Conclusions:**

The present data collection tool for fatal road traffic accidents was more complete and accurate than the current data collection tool and was confirmed to be a valid tool and suitable to be used in the Iranian Integrated Road Traffic Injury Registry and similar settings.

**Keywords:**

Injury surveillance, Registry; Road Traffic Injuries; Forensic medicine; Data collection tool; Validity

